# Statistical tests to identify appropriate types of nucleotide sequence recoding in molecular phylogenetics

**DOI:** 10.1186/1471-2105-15-S2-S8

**Published:** 2014-01-31

**Authors:** Victor A Vera-Ruiz, Kwok W Lau, John Robinson, Lars S Jermiin

**Affiliations:** 1 School of Mathematics and Statistics, University of Sydney, NSW 2006, Australia; 2 School of Biological Sciences, University of Sydney, NSW 2006, Australia; 3 CSIRO Computational Informatics, Floreat, WA, 6014, Australia; 4 CSIRO Ecosystem Sciences, Canberra, ACT 2601, Australia

**Keywords:** Phylogeny, Markov model, stationarity, homogeneity,reversibility, recoding, lumping, nucleotides, primates

## Abstract

**Background:**

Under a Markov model of evolution, recoding, or lumping, of the four nucleotides into fewer groups may permit analysis under simpler conditions but may unfortunately yield misleading results unless the evolutionary process of the recoded groups remains Markovian. If a Markov process is lumpable, then the evolutionary process of the recoded groups is Markovian.

**Results:**

We consider stationary, reversible, and homogeneous Markov processes on two taxa and compare three tests for lumpability: one using an *ad hoc *test statistic, which is based on an index that is evaluated using a bootstrap approximation of its distribution; one that is based on a test proposed specifically for Markov chains; and one using a likelihood-ratio test. We show that the likelihood-ratio test is more powerful than the index test, which is more powerful than that based on the Markov chain test statistic. We also show that for stationary processes on binary trees with more than two taxa, the tests can be applied to all pairs. Finally, we show that if the process is lumpable, then estimates obtained under the recoded model agree with estimates obtained under the original model, whereas, if the process is not lumpable, then these estimates can differ substantially. We apply the new likelihood-ratio test for lumpability to two primate data sets, one with a mitochondrial origin and one with a nuclear origin.

**Conclusions:**

Recoding may result in biased phylogenetic estimates because the original evolutionary process is not lumpable. Accordingly, testing for lumpability should be done prior to phylogenetic analysis of recoded data.

## Introduction

When nucleotides intentionally are recoded to a 3- or 2-state alphabet in order to focus on a subset of the possible types of substitutions (e.g., transversions [1-3]) or reduce compositional heterogeneity [4], it is no longer appropriate to use model-based phylogenetic methods that rely solely on time-reversible, 4-state Markov models. Instead, one needs to use a 3- or 2-state Markov model to approximate the evolutionary processes for the recoded sequence data. This requirement was first realised by Phillips and Penny [5], who used a time-reversible 2-state Markov model [6] to analyse *RY*-recoded nucleotide sequences, and Gibson et al. [7], who developed a time-reversible 3-state Markov model to analyse *Y*-recoded nucleotide sequences. Before these studies, other investigators had used *RY*-recoded nucleotide sequences to infer the evolutionary relationships among mammals [1-3] and among bacteria [4].

Recoding of nucleotides and/or amino acids has been used repeatedly in recent phylogenetic studies [8-31]. However, the mathematical principles underpinning the recoding of nucleotides or amino acids have not yet been adequately examined. For example, it is not yet known whether the Markovian property is maintained after recoding and how this should be tested [32]. Without this knowledge, we may run the risk of using a promising procedure in a manner that turns out to be inappropriate for the data.

In this paper, we take a first step by considering tests for lumpability in a Markov model of evolution for pairs of homologous nucleotide sequences (we are aware of only one paper in the phylogenetic literature where the term lumpability is used [33], but there it was used in a different context). We only consider nucleotides but believe our tests could be generalized to encompass amino acids as well. We then illustrate the performance of our tests for lumpability using simulated and real data, and show that recoding of nucleotides should be used with caution when analysing DNA phylogenetically.

## Methods

### The theoretical basis for recoding nucleotides

Let $S=\left\{A,C,G,T\right\}$ be the set of nucleotides, and let $S^{\prime }$ be a partition of  S such that $S^{\prime }=\left\{S_{\text{1}},...,S_{q}\right\}$, where *q <*4. Then  S is reduced by grouping, or lumping, some of the original states (i.e., *A*, *C*, *G*, and *T*) into one or two new states (i.e., *R*, *Y*, *S*, *W*, *M*, *K*, *B*, *D*, *H*, and *V *)--in molecular phylogenetics, this procedure has been called *recoding *[34]. Table 1 presents the 13 possible recoding schemes and partitions of $S^{\prime }$ using notation established by the NC-IUB [35]. The 13 recoding schemes fall into three major grouping categories, as shown in Table 1.

**Table 1:** The 13 ways of reducing a 4-letter state space ( S) to a 3- or 2-letter state space $S^{\prime }$.

Nucleotide-grouping Subsets	Recoding notation	Resulting $S^{\prime }$	Major grouping category
{{*A*, *G*}, *C*, *T*}	*R*	{*R*, *C*, *T*}	{2 : 1 : 1}
{*A*, *G*, {*C*, *T*}}	*Y*	{*A*, *G*, *Y*}	
{*A*, {*C*, *G*}, *T*}	*S*	{*A*, *S*, *T*}	
{*C*, *G*, {*A*, *T*}}	*W*	{*C*, *G*, *W*}	
{*A*, *C*, {*G*, *T*}}	*M*	{*M*, *G*, *T*}	
{*C*, {*A*, *G*, *T*}}	*K*	{*A*, *C*, *K*}	

{*A*, {*C*, *G*, *T*}}	*B*	{*A*, *B*}	{3 : 1}
{*C*, {*A*, *G*, *T*}}	*D*	{*C*, *D*}	
{*G*, {*A*, *C*, *T*}}	*H*	{*G*, *H*}	
{*T*, {*A*, *C*, *G*}}	*V*	{*T*, *V*}	

{{*A*, *G*}, {*C*, *T*}}	*RY*	{*R*, *Y*}	{2 : 2}
{{*A*, *T*}, {*C*, *G*}}	*SW*	{*S*, *W*}	
{{*A*, *C*}, {*G*, *T*}}	*KM*	{*K*, *M*}	

### The evolutionary process for two homologous nucleotide sequences

Consider two nucleotide sequences, A and B, each with *n *independently evolving sites, which have diverged under Markovian conditions from their common ancestor on a rooted, 2-tipped tree. Let *π*_0 _denote the initial probability vector of the nucleotide frequencies, such that $\pi_{0}^{T}=\left(\pi_{0_{1}},\pi_{0_{2}},\pi_{0_{3}},\pi_{0_{4}}\right)$, where, for convenience of notation, we will use the subscripts 1, 2, 3, 4 to denote *A*, *G*, *C*, *T *. Over each edge of this tree, there is a substitution process, *X*(*t*) and *Y*(*t*), respectively, described by the transition probabilities

$$P_{ij}^{X}\left(t\right)=P\left[X\left(t\right)=j\text{|}X\left(0\right)=i\right]$$

and

$$P_{ij}^{Y}\left(t\right)=P\left[Y\left(t\right)=j\text{|}Y\left(0\right)=i\right].$$

Let *f_ij_*(*t*) denote the theoretical joint probability of a site being in state *i *in A and state *j *in B at time *t*:

(1) $$f_{ij}\text{(}t\text{)}=P[X\left(t\right)=i,Y\left(t\right)=j\text{|}X\left(0\right)=Y\left(0\right)\text{]}.$$

Now, let **F**(*t*) = {*f_ij_*(*t*)} denote the joint probability matrix and let **P***^X^*(*t*) and **P***^Y^*(*t*) denote the transition probability matrices of *X*(*t*) and *Y*(*t*). In practice, the two matrices cannot be identified from **F**(*t*) without some assumptions about the evolutionary processes of the sequences A and B. We assume that the processes are globally stationary, reversible, and homogeneous (SRH) (for definitions, see [36-39], and, in more detail, [40]). Given these assumptions, take **P***^X^*(*t*) = **P***^Y^*(*t*) = **P**(*t*) and, if *π_X _*and *π_Y _*denote the equilibrium probability distributions of the processes *X*(*t*) and *Y*(*t*), take *π_X _*= *π_Y _*= *π*_0 _= *π *and write Π = *diag*(*π*). Then, from (1), we get **F**(*t*) = **P**(*t*)*^T^*Π**P**(*t*). The transition probability matrix can be expressed by an instantaneous rate matrix **R**, such that **P**(*t*) = *e*^**R***t*^, where *R_ij _≥ *0 for *i ≠ j*, $R_{ii}=-\sum_{i\neq j}^{4}R_{ij},$ and *π^T^***R **= **0***^T^*, where *π^T ^*is the equilibrium distribution of **R **[40]. Furthermore, the instantaneous rate matrix can take the form **R **= **S**Π, where **S **is a symmetric matrix with *s_ij _≥ *0 for *i ≠ j*, and $s_{ii}=-\sum_{i\neq j}^{4}s_{ij}\pi_{j}\text{/}\pi_{i}$[40]. The matrices **R **and **P**(*t*) can be written in terms of the eigenvector decomposition of Π^1*/*2^**S**Π^1*/*2^ = **L**Λ**L***^T^*. In other words

(2) $$R={\text{Π}}^{-1/2}L\text{Λ}L^{T}{\text{Π}}^{1/2}$$

and

(3) $$P\left(t\right)={\text{Π}}^{-1/2}Le^{\text{Λ}t}L^{T}{\text{Π}}^{\text{1}/\text{2}},$$

where **Λ **is a diagonal matrix with columns containing the eigenvalues of **Π**^1*/*2^**SΠ**^1*/*2^ and **L **is a matrix with columns containing its right eigenvectors. The joint probability matrix is then symmetric and

(4) $$F\left(t\right)={\text{Π}}^{\text{1}/\text{2}}Le^{\text{2}\text{Λ}t}L^{T}{\text{Π}}^{\text{1}/\text{2}}.$$

Note that under these assumptions, there are only nine free parameters to be estimated: six free parameters for the off-diagonal elements of **S **(define *s^T ^*= (*s*_12_, *s*_13_, *s*_14_, *s*_23_, *s*_24_, *s*_34_)) and three free parameters for *π *(because $\pi_{4}=1-\sum_{i=1}^{3}\pi_{i}$). The time *t *can be fixed at 1 since modifying it is equivalent to modifying the *s*-parameters.

Let **N **denote the 4 *× *4 divergence matrix for A and B, such that **N **= {*n_ij_*}, where *n_ij _*represents the number of homologous sites that are in state *i *in A and state *j *in B. Under the model, the vector of elements of **N **has a multinomial distribution with parameters *n *and **F**(*t*); its expected value is thus *E*(**N**) = *n***F**(*t*). Because the parameters *s *and *π *are in a one-to-one relation with the elements of **F**, the maximum-likelihood estimates of *s *and *π *can be obtained from the eigenvector decomposition of $\hat{F}\left(t\right)=\frac{1}{2n}\left(N+N^{T}\right)$, then

(5) $$\hat{\text{Π}}=diag\left(\hat{F}\left(1\right)1\right)$$

and

(6) $$\hat{S}={\hat{\text{Π}}}^{-1/2}\hat{L}\hat{\text{Λ}}{\hat{L}}^{T}{\hat{\text{Π}}}^{-1/2},$$

where $\hat{L}$ and $\hat{\text{Λ}}$ are obtained from ${\hat{\text{Π}}}^{-1/)2}\hat{F}\left(1\right){\hat{\text{Π}}}^{-1/)2}=\hat{L}e^{2\hat{\text{Λ}}}{\hat{L}}^{T}$.

### Lumpable Markov chains

The following probabilities can be defined for any given $S^{\prime }=\left\{S_{\text{1}},\cdot \cdot \cdot ,S_{q}\right\}$, where *q <*4, such that a *lumped process*, $X^{\prime }\left(t\right)$, with a smaller number of states, is generated with transition probabilities

(7) $$\begin{array}{c} {P^{\prime }}_{kl}\left(t\right)=P\left[X^{\prime }\left(t\right)=l\text{|}X^{\prime }\left(0\right)=k\right]\\ \;\;\;\;\;\;\;\;\;\;\;\;\;\;=P\left[X\left(t\right)\in S_{l}\text{|}X\left(0\right)\in S_{k}\right], \end{array}$$

and initial probabilities $\pi^{\prime }=P\left[X^{\prime }\left(0\right)=k\right]=P\left[X\left(0\right)\in S_{k}\right]$.

By definition [41,42], a Markov process is lumpable if, for every starting vector *π*, the lumped process, defined in (7), is a Markov chain whose transition probabilities do not depend on the choice of *π*. A necessary and sufficient condition for *X'*(*t*) to be lumpable with respect to a partition $S^{\prime }$ is that for every pair of subsets, $S_{k}$ and $S_{l}$, $\sum_{j\in S_{l}}P_{ij}\left(t\right)$, has the same value for every state *i *in $S_{k}$[41,42]. Accordingly, if *X'*(*t*) is lumpable, then the transition probabilities for *X'*(*t*) for any given pair of subsets in $S^{\prime }$ are

$$P_{kl}^{\prime }\left(t\right)=\underset{j\in S_{l}}{\sum }P_{ij}\left(t\right),\;\text{for}\;\text{any}\;i\in S_{k}.$$

If the Markov chain is lumpable, the lumped transition matrix **P**'(*t*) can be expressed as a matrix function of **P**(*t*) as follows:

$$P^{\prime }\left(t\right)=UP\left(t\right)V,$$

where **V **is a 4 *× q *matrix, where *q *is the number of states in the lumped process, such that the *l*-th column of **V **is a vector with 1's in the components corresponding to states in *S_l _*and 0's otherwise, and

$$U={\left(V^{T}\text{Π}V\right)}^{-1}V^{T}\text{Π},$$

is a *q × *4 matrix whose *k*-th row is a probability vector with non-zero elements corresponding to the states in $S_{k}$. A useful necessary and sufficient condition for lumpability [41,42] is

(8) VUP(t)V=P(t)V.

In the case of nucleotides, the second column of Table 2 gives the conditions required for lumpability of four nucleotides.

**Table 2:** Conditions required for a 4-state Markovian process to be lumpable (in terms of *s *and *π*), and transformations to obtain $\tilde{\pi }$ and $\tilde{s}$ such that the lumpability holds.

$S^{\prime }$	Lumpability conditions	($\tilde{\pi }$,$\tilde{s}$)
{{*A*, *G*}, *C*, *T*}	*s*_12 _= *s*_23_ *s*_14 _= *s*_34_	${\tilde{s}}_{\text{13}}=\;{\tilde{s}}_{\text{23}}=\left({ŝ}_{\text{13}}+{ŝ}_{\text{23}}\right)/\text{2}$ ${\tilde{s}}_{\text{14}}=\;{\tilde{s}}_{\text{34}}=\left({ŝ}_{\text{14}}+{ŝ}_{\text{34}}\right)/\text{2}$

{*A*, *G*, {*C*, *T*}}	*s*_12 _= *s*_14_ *s*_23 _= *s*_34_	${\tilde{s}}_{\text{12}}=\;{\tilde{s}}_{14}=\left({ŝ}_{\text{12}}+{ŝ}_{\text{14}}\right)/\text{2}$ ${\tilde{s}}_{\text{23}}=\;{\tilde{s}}_{\text{34}}=\left({ŝ}_{\text{23}}+{ŝ}_{\text{34}}\right)/\text{2}$

{*A*, {*C*, *G*}, *T*}	*s*_12 _= *s*_13_ *s*_24 _= *s*_34_	${\tilde{s}}_{\text{12}}=\;{\tilde{s}}_{\text{13}}=\left({ŝ}_{\text{12}}+{ŝ}_{\text{13}}\right)/\text{2}$ ${\tilde{s}}_{\text{24}}=\;{\tilde{s}}_{\text{34}}=\left({ŝ}_{\text{24}}+{ŝ}_{\text{34}}\right)/\text{2}$

{*C*, *G*, {*A*, *T*}}	*s*_12 _= *s*_24_ *s*_13 _= *s*_34_	${\tilde{s}}_{\text{12}}=\;{\tilde{s}}_{\text{24}}=\left({ŝ}_{\text{12}}+{ŝ}_{\text{24}}\right)/\text{2}$ ${\tilde{s}}_{\text{13}}=\;{\tilde{s}}_{\text{34}}=\left({ŝ}_{\text{13}}+{ŝ}_{\text{34}}\right)/\text{2}$

{{*A*, *C*}, *G*, *T*}	*s*_13 _= *s*_23_ *s*_14 _= *s*_24_	${\tilde{s}}_{\text{13}}=\;{\tilde{s}}_{\text{23}}=\left({ŝ}_{\text{13}}+{ŝ}_{\text{23}}\right)/\text{2}$ ${\tilde{s}}_{\text{14}}=\;{\tilde{s}}_{\text{24}}=\left({ŝ}_{\text{14}}+{ŝ}_{\text{24}}\right)/\text{2}$

{*A*, *C*, {*G*, *T*}}	*s*_13 _= *s*_14_ *s*_12 _= *s*_13_	${\tilde{s}}_{\text{13}}=\;{\tilde{s}}_{\text{14}}=\left({ŝ}_{\text{13}}+{ŝ}_{\text{14}}\right)/\text{2}$ ${\tilde{s}}_{\text{23}}={\tilde{s}}_{\text{24}}=\left({ŝ}_{\text{23}}+{ŝ}_{\text{24}}\right)/\text{2}$

{*A*, {*C*, *G*, *T*}}	*s*_12 _= *s*_13_ *s*_13 _= *s*_14_	${\tilde{s}}_{\text{12}}={\tilde{s}}_{\text{13}}=\;{\tilde{s}}_{\text{14}}=\left({ŝ}_{\text{12}}+{ŝ}_{\text{13}}+{ŝ}_{\text{14}}\right)/\text{3}$

{*C*, {*A*, *G*, *T*}}	*s*_12 _= *s*_23_ *s*_23 _= *s*_24_	${\tilde{s}}_{\text{12}}={\tilde{s}}_{\text{23}}=\;{\tilde{s}}_{\text{24}}=\left({ŝ}_{\text{12}}+{ŝ}_{\text{23}}+{ŝ}_{\text{24}}\right)/\text{3}$

{*G*, {*A*, *C*, *T*}}	*s*_13 _= *s*_23_ *s*_23 _= *s*_34_	${\tilde{s}}_{\text{13}}={\tilde{s}}_{\text{23}}=\;{\tilde{s}}_{\text{34}}=\left({ŝ}_{\text{13}}+{ŝ}_{\text{23}}+{ŝ}_{\text{34}}\right)/\text{3}$

{*T*, {*A*, *C*, *G*}}	*s*_14 _= *s*_24_ *s*_24 _= *s*_34_	${\tilde{s}}_{\text{14}}={\tilde{s}}_{\text{24}}=\;{\tilde{s}}_{\text{34}}=\left({ŝ}_{\text{14}}+{ŝ}_{\text{24}}+{ŝ}_{\text{34}}\right)/\text{3}$

{{*A*, *G*}, {*C*, *T*}}	*s*_12_*π*_2 _+ *s*_14_*π*_4 _= *s*_23_*π*_2_+ *s*_34_*π*_4_ *s*_12_*π*_1 _+ *s*_23_*π*_3 _= *s*_14_*π*_1_+ *s*_34_*π*_3_	${\tilde{s}}_{23}=\frac{{\tilde{s}}_{12}\left({\hat{\pi }}_{2}{\hat{\pi }}_{3}-{\hat{\pi }}_{1}{\hat{\pi }}_{4}\right)\;+\;{\tilde{s}}_{14}{\hat{\pi }}_{4}\left({\hat{\pi }}_{1}+{\hat{\pi }}_{3}\right)\;}{{\hat{\pi }}_{3}\left({\hat{\pi }}_{2}+{\hat{\pi }}_{4}\right)\;}$ ${\tilde{s}}_{34}=\frac{{\tilde{s}}_{12}{\hat{\pi }}_{1}+{\tilde{s}}_{23}{\hat{\pi }}_{3}-{\tilde{s}}_{14}{\hat{\pi }}_{1}}{{\hat{\pi }}_{3}}$

{{*A*, *T*}, {*C*, *G*}}	*s*_12_*π*_2 _+ *s*_13_*π*_3 _= *s*_24_*π*_2_+ *s*_34_*π*_3_ *s*_12_*π*_1 _+ *s*_24_*π*_4 _= *s*_13_*π*_1_+ *s*_34_*π*_4_	${\tilde{s}}_{13}=\frac{{\tilde{s}}_{12}\left({\hat{\pi }}_{1}{\hat{\pi }}_{3}-{\hat{\pi }}_{2}{\hat{\pi }}_{4}\right)\;+\;{\tilde{s}}_{24}{\hat{\pi }}_{4}\left({\hat{\pi }}_{2}+{\hat{\pi }}_{3}\right)\;}{{\hat{\pi }}_{3}\left({\hat{\pi }}_{1}+{\hat{\pi }}_{4}\right)\;}$ ${\tilde{s}}_{34}=\frac{{\tilde{s}}_{12}{\hat{\pi }}_{2}+{\tilde{s}}_{13}{\hat{\pi }}_{3}-{\tilde{s}}_{24}{\hat{\pi }}_{2}}{{\hat{\pi }}_{3}}$

{{*A*, *C*}, {*G*, *T*}}	*s*_13_*π*_3 _+ *s*_14_*π*_4 _= *s*_23_*π*_3 _+ *s*_24_*π*_4_ *s*_13_*π*_1 _+ *s*_23_*π*_2 _= *s*_14_*π*_1 _+ *s*_24_*π*_4_	${\tilde{s}}_{23}=\frac{{\tilde{s}}_{13}\left({\hat{\pi }}_{2}{\hat{\pi }}_{3}-{\hat{\pi }}_{1}{\hat{\pi }}_{4}\right)\;+\;{\tilde{s}}_{14}{\hat{\pi }}_{4}\left({\hat{\pi }}_{1}+{\hat{\pi }}_{2}\right)\;}{{\hat{\pi }}_{2}\left({\hat{\pi }}_{3}+{\hat{\pi }}_{4}\right)\;}$ ${\tilde{s}}_{24}=\frac{{\tilde{s}}_{13}{\hat{\pi }}_{1}+{\tilde{s}}_{23}{\hat{\pi }}_{2}-{\tilde{s}}_{14}{\hat{\pi }}_{1}}{{\hat{\pi }}_{2}}$

We note that under certain conditions, such as those considered by the JC [43] and F81 [44] models, all recodings are lumpable. Conditions under which recoding of nucleotides are possible for the K2P [45] and HKY [46] models are given in Table 3.2 of [32].

### Tests for lumpability

We consider three possible tests: An *ad hoc *test based on a parametric bootstrap for an index of departure from the lumpability condition [32]; a test based on a test for lumpability in Markov chains [47]; and a likelihood-ratio test.

#### Index test

From (8), if a Markov process is lumpable, then

M=VUP(t)V-P(t)V

should have all elements zero. Consider the index proposed in [32]:

(9) $$\eta ={\left(\underset{i,j}{\sum }m_{ij}^{2}\right)}^{1/2},\text{where }m_{ij}=M.$$

It is clear that *η ≥ *0, with *η *being 0 only under lumpable Markovian processes. Then, the hypothesis that the Markov process is lumpable is equivalent to the hypothesis *H*_0 _: *η *= 0. From the observed divergence matrix, **N **of two homologous sequences, assuming a SRH Markovian model of evolution, an estimate $\hat{\eta }$ can be used as a test statistic for *H*_0_, where

$$\hat{\eta }={\left(\underset{i,j}{\sum }{\hat{m}}_{ij}^{2}\right)}^{1/2},$$

and $\hat{M}=VU\hat{P}\left(1\right)V-\hat{P}\left(1\right)V$ for $\hat{P}\left(1\right)=\text{exp}\left(\hat{S}\hat{\text{Π}}\right)$.

The distribution of $\hat{\eta }$ is unknown, so we propose an approximation to it that is based on the parametric bootstrap. The estimated vectors $\hat{\pi }$ and ŝ  do not necessarily satisfy the conditions for lumpability, so we obtain $\tilde{\pi }$ and $\tilde{s}$ using the relevant equations from the third column of Table 2 as estimates that do satisfy the lumpability condition. Once the $\tilde{\pi }$ and $\tilde{s}$ vectors are calculated, a procedure similar to that shown in (2), (3) and (4) is carried out such that the matrices $\tilde{R}$, $\tilde{P}$(1), and $\tilde{F}\left(1\right)$ are generated under the lumpability conditions. Now *B *matrices can be generated by simulation under conditions of lumpability, where we take $N_{b}^{*}$, with *b *∈ {1, *⋯*, *B*}, to be independent and multinomial with parameters *n *and $\tilde{F}\left(1\right)$. From each of these simulated samples, we calculate $F_{b}^{*}\text{(1)}=\left(N_{b}^{*}+N_{b}^{*T}\right)\text{/2}$, ${\text{Π}}_{b}^{*}$ and $S_{b}^{*}$ from $F_{b}^{*}$, as in (5) and (6), and then $P_{b}^{*}\left(1\right)=\text{exp}\left(S_{b}^{*}{\text{Π}}_{b}^{*}\right),M_{b}^{*}={VUP}_{b}^{*}\left(1\right)V-P_{b}^{*}\left(1\right)V$, and

$$\eta_{b}^{*}={\left(\sum_{i,j}{\hat{m}}_{bij}^{{{}_{*}}^{{}_{2}}}\right)}^{1/2}.$$

The true *P*-value is then the probability that we obtain a value as large as or larger than the observed $\hat{\eta }$, so a bootstrap approximation to this *P*-value is the proportion of $\eta_{i}^{*},...,\eta_{B}^{*}$ exceeding $\hat{\eta }$.

#### Markov chain test

A *χ*^2^ test to determine whether a Markov chain is lumpable with respect to a partition $S^{\prime }$ is available [47]. The test is based on the comparison of observed transition frequencies to their respective theoretical counterparts under the null hypothesis that the chain is lumpable. The approach does not make any assumption about reversibility or stationarity of the process. The authors used a matrix of transition counts, {*n_ij_*}, to estimate the transition probabilities *p_ij_*, where *n_ij _*represents the number of transitions into state *j *from state *i *in one step, so the number of steps in the Markov chain is *n_••_*, where the subscript *• *indicates summation. Now, if we start from our divergence matrix **N**, where *n_ij _*represents the number of sites that are in state *i *for sequence A and state *j *in sequence B, and the SRH assumptions are kept, either A or B can be assumed to be the original sequence at time 0, whereas the other one can be assumed to be the observed sequence at time 2 (since we took the edge lengths to be 1). Take A as the ancestral sequence, then the divergence matrix **N **has the same properties as a transition count matrix, and we can proceed as described in [47]. A transition probability from *i *to $S_{l}$ is

$$g_{il}=\underset{j\in S_{l}}{\sum }P_{ij}\left(2\right),$$

where *l *= 1, *..*., *q*. From the definition of a lumpable process (7), if the Markovian process is lumpable with respect to $S^{\prime }$, then

$$g_{il}=\underset{i\in S_{k}}{\sum }\underset{j\in S_{l}}{\sum }P_{ij}\left(2\right)/\gamma_{k},$$

where *γ_k _*is the number of states that are part of the subset $S_{k}$, and *k *= 1, *..*., *q*. Therefore, $g_{il}=P_{kl}^{\prime }\left(\text{2}\right)$ if the process is lumpable and the null hypothesis of lumpability can be expressed as *H*_0 _: $g_{il}=P_{kl}^{\prime }\left(\text{2}\right)$ for all *i *∈ $S_{k}$. Given the divergence matrix, **N**, estimates *g_il _*and $P_{kl}^{\prime }\left(\text{2}\right)$ are

$${ĝ}_{il}=\underset{j\in S_{l}}{\sum }\frac{n_{ij}}{n_{i∙}}$$

and

$${\hat{P}}_{kl}^{\prime }\left(2\right)=\frac{n_{kl}^{\prime }}{n_{k∙}^{\prime }}=\frac{\sum_{i\in S_{k}}\sum_{j\in S_{l}}n_{ij}}{\sum_{i\in S_{k}}n_{i∙}},$$

where $N^{\prime }=\left\{n_{kl}^{\prime }\right\}$ is the divergence matrix of the recoded nucleotide sequences. Jernigan and Baran [47] obtained the test statistic

$$T=\overset{4}{\underset{i=1}{\sum }}\overset{q}{\underset{l=1}{\sum }}\frac{{\left(o_{il}-e_{il}\right)}^{2}}{e_{il}},$$

where

$$o_{il}=\overset{}{\underset{j\in S_{l}}{\sum }}n_{ij}$$

and

$$e_{il}=\frac{n_{i∙}n_{kl}^{\prime }}{n_{k∙}^{\prime }},$$

and showed (by pointing out that *o_il _− e_il _*are a stack of *q *tables of size 4 *× γ_l _*of mean-corrected multinomials with row and column sums equal to zero) that the test statistic is distributed under *H*_0 _as a *χ*^2^ variable with $\left(q-\text{1}\right)\sum_{k=1}^{q}\left(\gamma_{k}-\text{1}\right)$ degrees of freedom, if all cells are non-zero. In the case considered here, the degrees of freedom for any of the recoding schemes is 2.

#### Likelihood-ratio test

Consider estimates $\left(\hat{\pi },\;ŝ\right)$ whose values maximize a log-likelihood function

$$L\left(\pi ,s\right)=\underset{i,j}{\sum }n_{ij}\text{ln}f_{ij}\left(\pi ,s\right),$$

where {*n_ij_*} = **N**, the observed divergence matrix, **F**_(*π*,*s*)_(1) = exp(**SΠ**) and {*f_ij_*(*π*, *s*)} = **F**_(*π*,*s*)_(1). These matrices are obtained as shown in (5) and (6). We also want to estimate (*π*, *s*) under the constraints imposed by the null hypothesis of lumpablity, *H*_0_. The constraints are given in the second column of Table 2. Then we can define the constrained estimates ($\tilde{\pi }$, $\tilde{s}$) to satisfy

$$L\left(\tilde{\pi },\;\tilde{s}\right)=\underset{\pi ,s\in H_{0}}{\text{max}}L\left(\pi ,s\right).$$

This maximization needs a new approach. We construct an orthogonal matrix, **A**, such that

As=y,

where *y *is the response constraint vector, defined such that two values of *y *are zero corresponding to the two constraints. The matrix **A **will, in the case of partitions into two groups of two, contain *π*, so to emphasise this possible dependence, write *y *= *g*(*s|π*). Also write *s *= **A**^−1^*y *= *g*^−1^(*y|π*). Then

$$L\left(\tilde{\pi },\;\tilde{s}\right)=\underset{\pi ,y}{\text{max}}L\left(\pi ,g^{-1}\left(y\text{|}\pi \right)\right).$$

The optimization process is done in two steps: the values of *s*, if dependent of *π*, are optimized given the original *π *set, then the *π *vector is optimized given the optimized values of *s*. This process is repeated until convergence is achieved.

From these two log-likelihood values, a log likelihood-ratio, *LR*, can be calculated with

$$LR=L\left(\hat{\pi },ŝ\right)-L\left(\tilde{\pi },\tilde{\text{s}}\right)$$

Under the null hypothesis of lumpability, 2 *× LR *is distributed as a *χ*^2^ variable with 2 degrees of freedom.

## Results

### Assessment of accuracy

In order to check the accuracy of the tests under the null hypothesis, Monte Carlo simulations were done from a set of parameters that meets the assumption of lumpability. The parameter vectors in this case were

$$\pi^{T}=\;\left(0.\text{1},0.\text{2},0.\text{3},0.\text{4}\right)$$

and

$$s^{T}=\;\left(0.\text{2},0.\text{25},0.\text{2},0.\text{2},0.\text{15},0.\text{2}\right).$$

The joint probability distribution was calculated by the steps given in (2), (3), and (4); then, assuming a nucleotide sequence of length *n *= 1500, 5000 divergence matrices were calculated by Monte Carlo simulations assuming that **N***_i _*is multinomial with parameters (*n*, **F**(1)) for *i *= 1, *. . *. , 5000.

**Figure 1:**
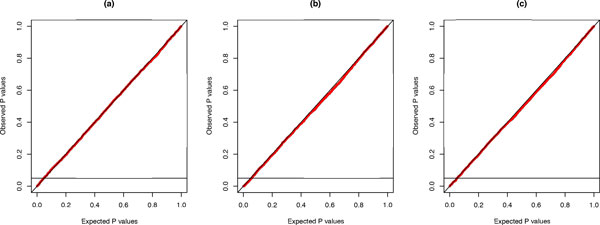
***PP*-plot for the three tests with respect to $S^{\prime }=\left\{R,Y\right\}$**. The observed *P*-values were calculated from 5000 Monte Carlo experiments for each test (i.e., the Index test (a), the Markov chain test (b), and the likelihood-ratio test (c)) and charted against the expected *P*-values.

The accuracy of each test for lumpability was verified using a *PP *plot displaying the distribution of observed *P*-values, obtained from each test, plotted against the expected *P*-values, obtained from the uniform distribution. The linear relationship between these two sets of *P*-values (Figure 1) confirms the accuracy of the tests.

### Comparisons of power

The power of each test was compared for each recoding scheme under non-lumpable conditions. To do this, we used *π^T ^*= (0.1, 0.2, 0.3, 0.4) and values of *s *that yield increasing values of *η*, as in (9), generated 3000 divergence matrices using Monte Carlo simulation, and then calculated the three test statistics and their corresponding *P*-values, using the procedures explained above, for each value of *η*. The power at the 5% level, is then equal to the proportion of observed *P*-values less than 0.05.

**Figure 2:**
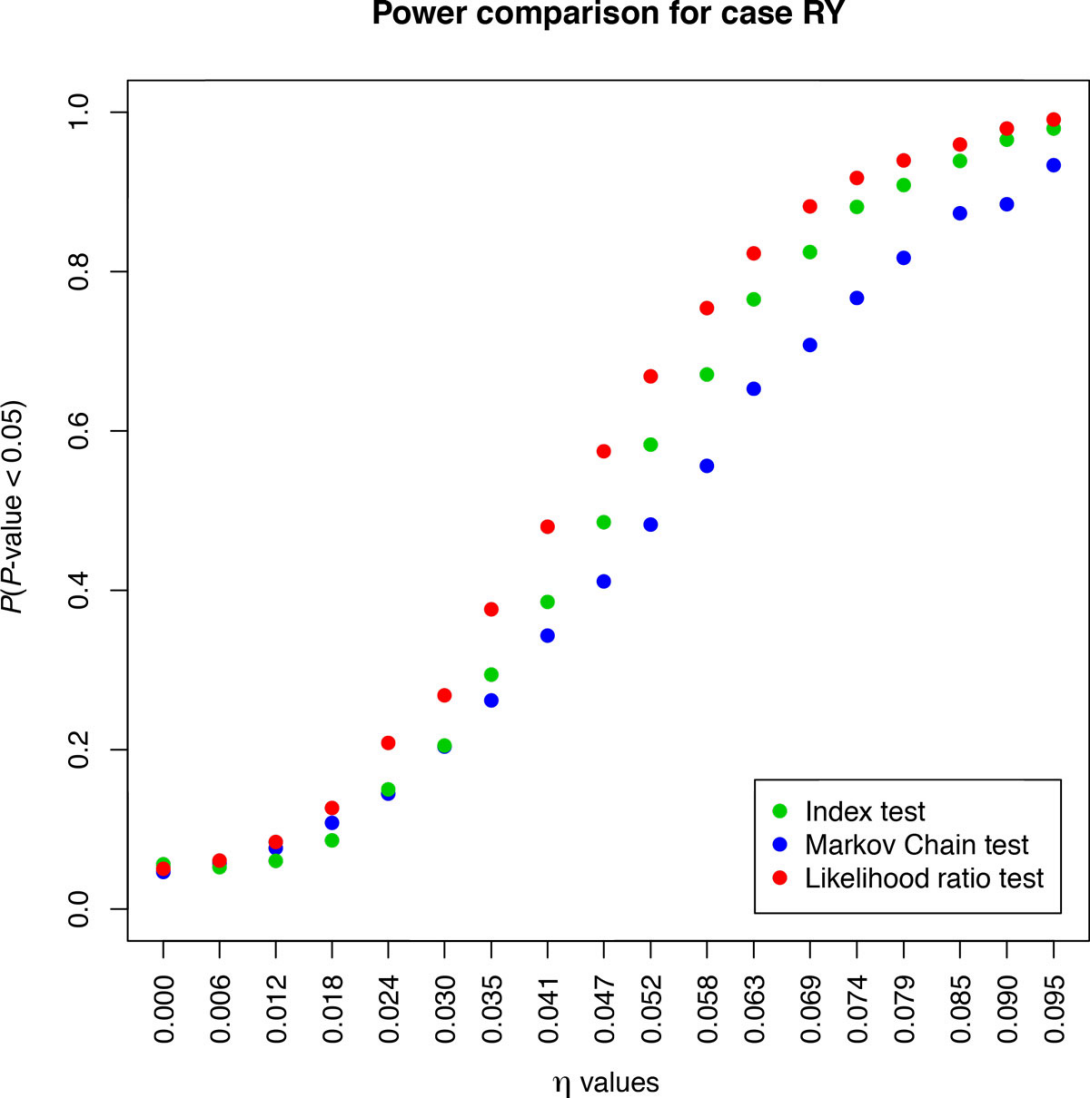
**Power curve for partition $S^{\prime }=\left\{R,Y\right\}$**. A total of 3000 divergence matrices were generated by Monte Carlo simulation for each triplet of points, corresponding to the three test statistics, and 500 parametric bootstraps were used during the calculation of *P*-values for the *η *index. The same *π*-vector (i.e., *π^T ^*= (0.1, 0.2, 0.3, 0.4)) was used for every triplet of points whereas the values in the *s*-vector were allowed to vary slightly for each point.

Figure 2 shows the power curves for *RY *recoding--similar power curves were obtained for the other 12 recoding schemes (results not shown). All of these results indicate that the likelihood-ratio test is the most powerful of the tests considered, followed by the Index test and, finally, by the Markov Chain test.

### Cases with more than 2 homologous sequences

For general cases involving more than 2 homologous sequences, we can test for lumpability in all pairs of sequences by using the methods described above under the assumption that the evolutionary process is SRH over the whole tree (i.e., the process is globally SRH). For example, in the case of an alignment with seven sequences, there will be 21 *P*-values. A *PP*-plot with these *P*-values should yield a straight line when the data are lumpable, and deviations from this expectation when the processes are not lumpable. However, the observed *P*-values are not independent, so we need to show that this condition is not cause for concern for the dots in a *PP*-plot to be on a straight line. We give a simplified argument taken from [48]. Consider a set of observed *P*-values *P*_1_, . . ., *P_n_*, which, if all the null hypotheses are true, are identically distributed as uniform random variables on (0, 1). Let *p *be any value between 0 and 1, let *I*(*P_j _< p*) take the value 1 if *P_j _< p *and 0 otherwise, and let $N_{p}=\sum_{j=1}^{n}I\left(P_{j}<p\right)$ be the number of observed *P*-values less than *p*. Then *E*(*I*(*P_j _< p*)) = *P *(*P_j _< p*) = *p *and so the expected number of *P*-values less than *p *is *E*(*N_p_*) = *np*. This implies that the plot of the observed *P*-values will lie approximately on a straight line. The dependence will cause some clustering of the observed *P*-values but the *PP*-plot will remain useful in indicating whether there is evidence against some of the hypotheses.

**Figure 3:**
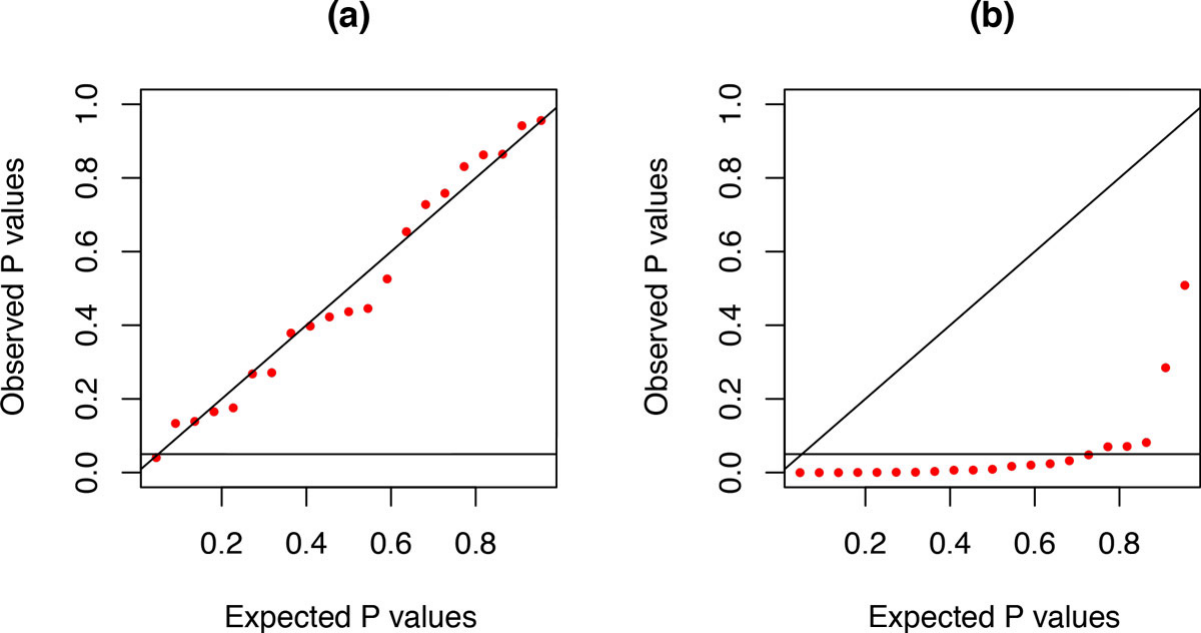
***PP*-plots for lumpability tests for simulated data**. *PP*-plots for the likelihood-ratio test, with respect to *RY *recoding, for the randomly-generated lumpable 7-taxon data (a) and the randomly-generated non-lumpable 7-taxon data (b). Sequences comprising 2000 sites were generated on the tree shown in Figure 4 under time-reversible conditions with sites evolving under independent and identical conditions ((a): *π^T ^*= (0.1, 0.2, 0.3, 0.4), and *s^T ^*= (0.20, 0.25, 0.20, 0.20, 0.15, 0.20); (b): *π^T ^*= (0.1, 0.2, 0.3, 0.4), and *s^T ^*= (0.50, 0.25, 0.20, 0.20, 0.15, 0.20)).

**Figure 4:**
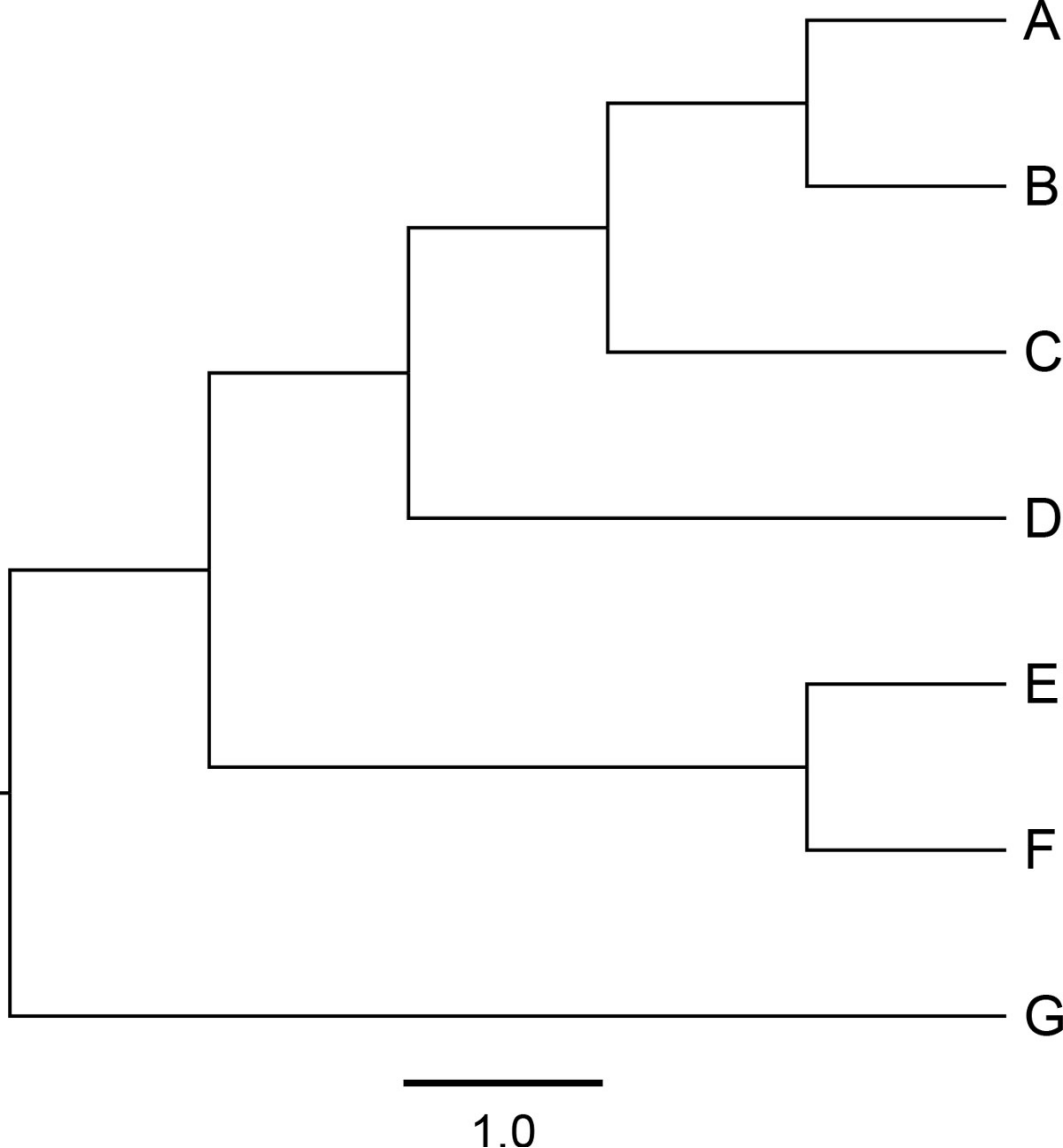
**Tree used to generate simulated data**. Tree used to generate the simulated data, which were then analysed to obtain the results shown in Figure 3. The scale bar corresponds to 1 time unit.

The *PP*-plots shown in Figure 3 were obtained from alignments of nucleotides generated under lumpable or non-lumpable conditions, with respect to *RY *recoding, on the tree shown in Figure 4 before being analysed using the likelihood-ratio test. From these two plots, it is clear that the test is able to identify cases where sequences have evolved under non-lumpable conditions.

### The effect of non-lumpability on phylogenetic estimates

If a process is lumpable with respect to a given recoding scheme (e.g., *RY*), then we can obtain **F***_q_*(*t*) = **V***^T^*P(*t*)**V** and from this, using (2, 3) and (4), we can further obtain the transition matrix of the process *X'*(*t*),

$$P_{q}\left(t\right)=\Pi_{q}^{-\text{1}/\text{2}}L_{q}{e^{\Lambda_{q}}}^{t}L_{q}^{T}\Pi_{q}^{\text{1}/\text{2}},$$

where **Π***_q _*= **V***^T^***ΠV **. If the process is not lumpable with respect to that recoding scheme, then *X'*(*t*) is not a Markov process and, although we can calculate **P***_q_*(*t*), it is *not *the transition matrix of the process *X'*(*t*).

In either case, the matrix **P***'*(*t*) = **UP**(*t*)**V **can be defined and, if the process *X*(*t*) is lumpable, then *P'*(*t*) = *P_q_*(*t*). On the other hand, if *X*(*t*) is not lumpable, the elements of **P***'*(*t*) are still given as $P_{kl}^{\prime }\text{(}t\text{)}=P\left(X^{\prime }\text{(}t\text{)}=l\text{|}X^{\prime }\text{(}0\text{)}\;\text{ = }\;k\right)$, but **P***'*(*t*) is no longer a transition matrix of *X'*(*t*). Conveniently, we can compare **P***'*(1), the true conditional probability matrix at *t *= 1, with **P***_q _*(1), the false transition matrix at *t *= 1, thus allowing us to examine the effect of non-lumpability.

**Figure 5:**
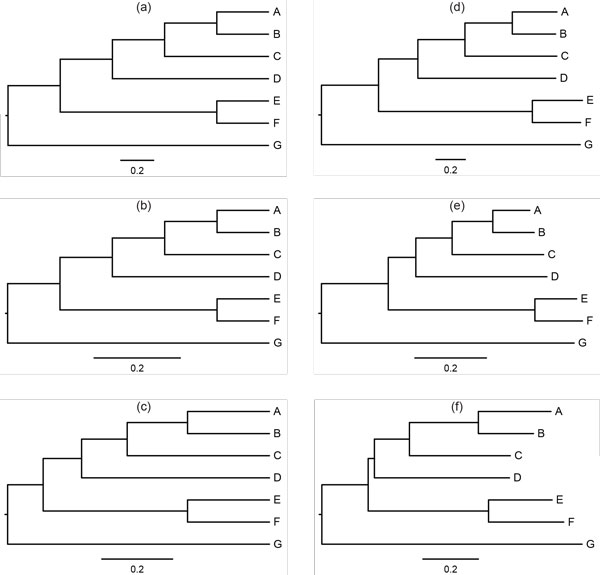
**Effect of recoding on phylogenetic estimates**. Panel (a) displays the tree that was used to generate alignments of 3000 nucleotides under a time-reversible 4-state Markov model. Panel (b) shows the corresponding tree with edge lengths adjusted according to the *RY*-recoding scheme. Panel (c) shows the corresponding tree with edge lengths adjusted according to the *KM*-recoding scheme. Panels (d), (e), and (f) present the corresponding results obtained by analysis of the data generated on the tree in panel (a). The scale bar corresponds to the expected number of substitutions $\left(\text{i}.\text{e}.,-\sum_{i=1}^{4}\pi_{i}r_{ii}\right)$.

Figure 5 illustrates the effect on phylogenetic estimates. Figure 5a shows the tree used to simulate alignments of 3000 nucleotides under a time-reversible 4-state Markov model with *π^T ^*= (0.2, 0.3, 0.2, 0.3) and *s^T ^*= (0.2, 0.1, 0.3, 0.3, 1.0, 0.2). As can be seen from the *π*- and *s*-vectors, the lumpable condition is met for *RY*-recoding but not for *KM*-recoding. Figures 5b and 5c display the corresponding tree with the edge lengths adjusted according to, respectively, the *RY*- and *KM*-recoding schemes.

Every edge in the tree obtained from the *RY*-recoded data is shorter than the corresponding edge in the tree obtained from the original data. However, because the original process was lumpable with respect to *RY*-recoding, the relative length of each edge in the two trees is the same, the difference being equal to a scale factor

$$\rho =\frac{\sum_{i=1}^{4}\pi_{i}r_{ii}}{\sum_{j=1}^{2}\pi_{2j\;}r_{2jj}}.$$

Every edge in the tree obtained from the *KM*-recoded data is also shorter than the corresponding edge in the tree obtained from the original data, but the relative length of each edge in the two trees differ, the reason being that the process generating the original data was not lumpable with respect to *KM*-recoding.

Figures 5d, 5e, and 5f show the corresponding results for the data generated by Monte Carlo simulation. The three trees display the same characteristics as those shown in Figures 5a, 5b, and 5c, while also showing some variation in the edge lengths that is due to the random nature of the data and the finite sample size. Hence, although recoding of nucleotides might be useful for a variety of reasons, using recoded data, without having tested for lumpability first, might lead to biased phylogenetic estimates.

### Example 1 -- Primate mitochondrial DNA

**Figure 6:**
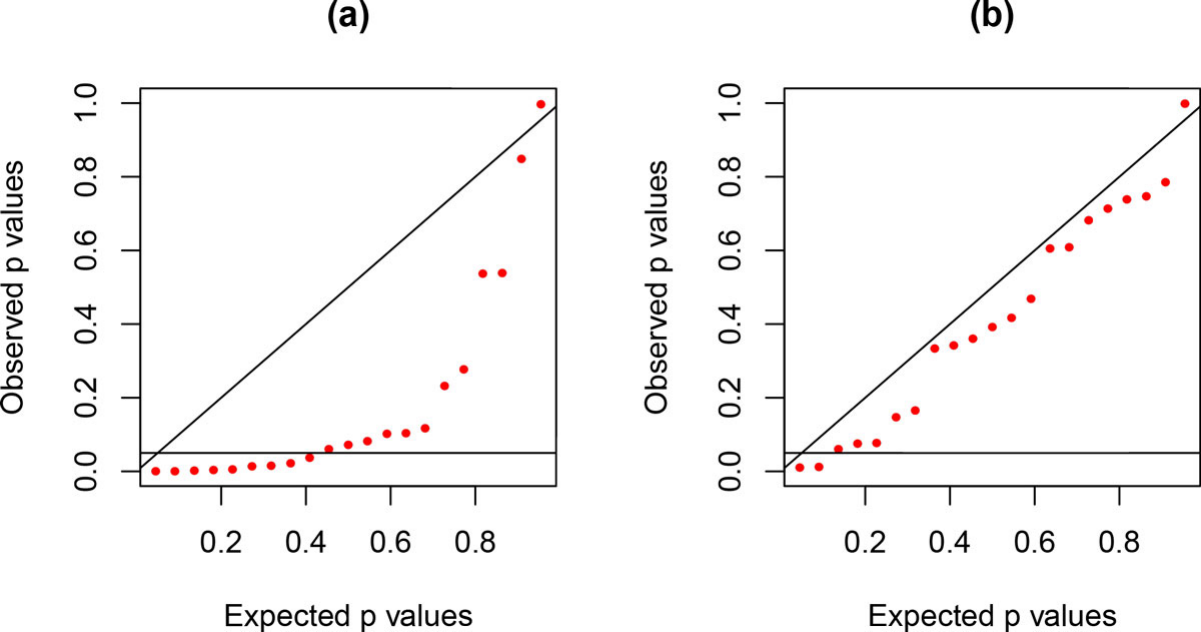
***PP*-plots for lumpability tests for hominoid mitochondrial data**. *PP*-plots for the likelihood-ratio tests for lumpability for hominoid data for *RY*-recoding (a) and for *AGY*-recoding (b), respectively.

In a previous study [37], a set of mitochondrial nucleotide sequences of hominoid origin were found to to fit the GTR model [49], implying that the data are consistent with evolution under globally SRH conditions. We applied the likelihood-ratio test to these data. Figure 6 shows the *PP*-plots from tests for lumpability for *RY *recoding, indicating non-lumpability, and *AGY *recoding, indicating lumpability.

### Example 2 -- Primate nuclear DNA

**Figure 7:**
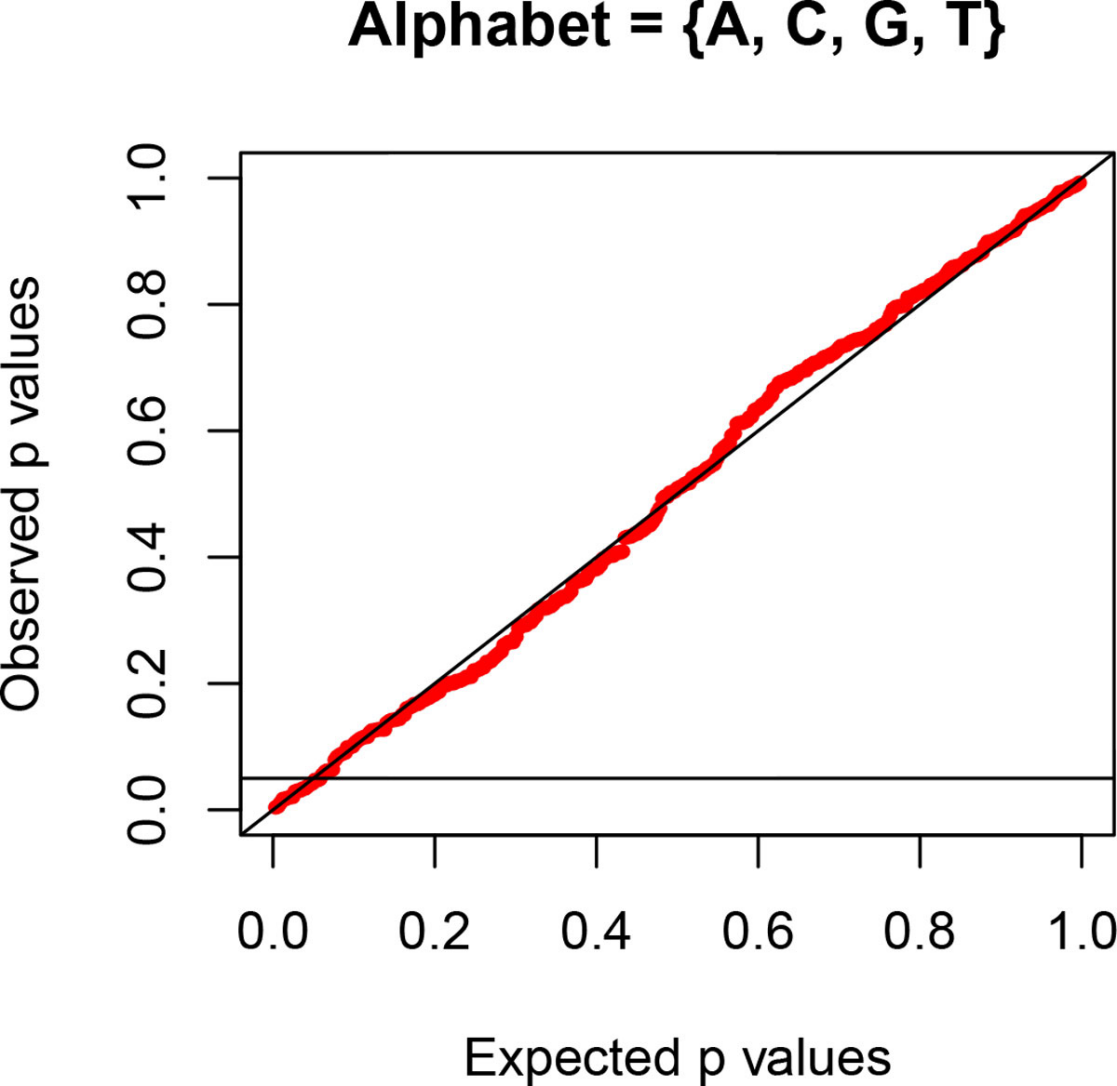
***PP*-plot for matched-pairs test of symmetry for primate nuclear data**. *PP*-plot demonstrating consistency with evolution under globally SRH conditions for the 4-state process.

**Figure 8:**
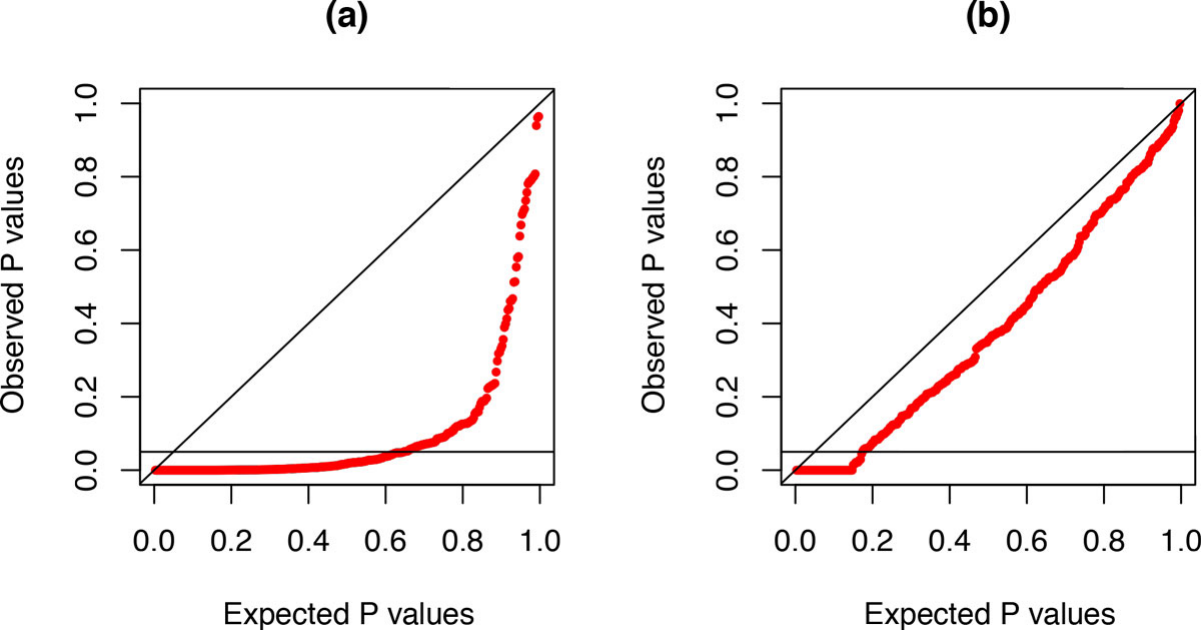
***PP*-plots for tests of lumpability for primate nuclear data**. *PP*-plots for lumpability for *RY*-recoding (a) and *SW*-recoding (b), respectively.

In a previous study [50], a ~9.3kb fragment of X chromosomal DNA was obtained from 26 species of primates and analysed phylogenetically using the HKY model. In so doing, the authors implicitly assumed evolution under globally SRH conditions. We wanted to apply the likelihood-ratio test to these data, so we obtained the same 26 sequences from GenBank [51], aligned them using MAFFT [52] (with the linsi option invoked), and, using SeaView [53], removed all columns with gaps and/or ambiguous characters. The resulting alignment contained 6913 sites from the 26 species. We then applied the matched-pairs test of symmetry [38] to the data to determine whether the sequences were consistent with evolution under globally SRH conditions. The *PP*-plot in Figure 7 clearly shows that the data are consistent with evolution under these conditions. Hence, it is appropriate to use our likelihood-ratio test to determine whether any of the recoding schemes would retain the Markovian properties of the original data. Figure 8 presents the *PP*-plots from the likelihood-ratio test for lumpability for *RY*-recoding, showing strong evidence against lumpability, and for the *SW*-recoding, which provided the least evidence against lumpability. It is evident that no recoding should be applied to these data.

## Conclusions

Bias in estimates of phylogenetic parameters can occur when recoding of nucleotides or amino acids is used to transform data associated with models of evolution, which are not lumpable with respect to the recoding scheme used. A test proposed in this paper, which is based on a likelihood-ratio test, can yield an indication of whether the same results for estimable parameters can be expected from fitting a given model of evolution and its recoded version to the data.

## Competing interests

The authors declare that they have no competing interests.

## Authors' contributions

LSJ and JR conceived the project. KWL carried out the pilot study, showing that the Index test was feasible. VAVR and JR developed the tests and wrote the manuscript with input from LSJ and KWL. VAVR wrote the codes and generated the numerical results. All authors have read and approved the manuscript.

## Declarations

The publication costs for this article were funded by resources made available to LSJ by CSIRO and JR by the School of Mathematics and Statistics, University of Sydney.

This article has been published as part of *BMC Bioinformatics *Volume 15 Supplement 2, 2014: Selected articles from the Twelfth Asia Pacific Bioinformatics Conference (APBC 2014): Bioinformatics. The full contents of the supplement are available online at http://www.biomedcentral.com/bmcbioinformatics/supplements/15/S2.
